# H-scan analysis of thyroid lesions

**DOI:** 10.1117/1.JMI.5.1.013505

**Published:** 2018-02-07

**Authors:** Gary R. Ge, Rosa Laimes, Joseph Pinto, Jorge Guerrero, Himelda Chavez, Claudia Salazar, Roberto J. Lavarello, Kevin J. Parker

**Affiliations:** a University of Rochester Medical Center, School of Medicine and Dentistry, Rochester, New York, United States; b Oncosalud, Departamento de Radiodiagnóstico, Lima, Perú; c Oncosalud, Unidad de Investigación Básica y Translacional, Lima, Perú; d Pontificia Universidad Católica del Perú, Laboratorio de Imágenes Médicas, Lima, Perú; e University of Rochester, Department of Electrical and Computer Engineering, Rochester, New York, United States

**Keywords:** ultrasonics, scattering, tissues, medical imaging

## Abstract

The H-scan analysis of ultrasound images is a matched-filter approach derived from analysis of scattering from incident pulses in the form of Gaussian-weighted Hermite polynomial functions. This framework is applied in a preliminary study of thyroid lesions to examine the H-scan outputs for three categories: normal thyroid, benign lesions, and cancerous lesions within a total group size of 46 patients. In addition, phantoms comprised of spherical scatterers are analyzed to establish independent reference values for comparison. The results demonstrate a small but significant difference in some measures of the H-scan channel outputs between the different groups.

## 1 Introduction

Ultrasound is the main imaging modality used to evaluate the thyroid for disease, aiding especially in the diagnosis, treatment, and management of thyroid cancers. Currently, the clinical utility of ultrasound includes assessing the structures of thyroid nodules, assisting in fine-needle aspiration biopsy of nodules, aiding in surgical planning, and monitoring the progression of thyroid cancers.^1^ Thyroid nodules are very common, with a prevalence of 2% to 6% by palpation and 19% to 35% with ultrasound.^2^ Although only about 5% of nodules are malignant, identifying malignancy early on is crucial. In addition, thyroid cancers are being discovered with increasing incidence due to use of ultrasound imaging. While the prognosis is usually better than with other malignant cancers, favorable outcomes are achieved only with coordinated therapies.^3^

Although size, shape, margin, echogenicity, and presence of microcalcifications may be helpful criteria for discriminating benign from malignant nodules, the sensitivities and specificities for some of these ultrasound criteria are still rather low.^4^^–^^6^ Efforts in ultrasound elastography have been shown to be promising in the differential diagnosis of thyroid cancers and have been compared with the best single method, fine needle aspiration biopsy. Moreover, these studies attempt to measure and utilize the structural properties of the nodules in differentiating between benign and malignant nodules.^5^^,^^7^^,^^8^^–^^10^ However, it has not been established that elastography techniques provide additional value in predicting malignancy of thyroid nodules.^11^

The structural differences between benign and malignant thyroid nodules may be characterized by the presence of different sized scatterers, corresponding to the structural changes seen in tissue histopathology. Certain chemical and molecular changes in the nucleus and cell morphology are seen in papillary thyroid carcinoma variants, and these changes have been characterized by scattering properties.^12^^–^^14^ In a population-based study by Smith-Bindman et al.,^15^ microcalcifications were associated with 38.2% of malignant nodules and 5.4% of benign nodules. Models for classifying microcalcifications as elastic scatterers under ultrasound have been studied previously.^16^ In addition, nodules may consist of colloid cysts or have very little fluid or colloid; the former is more likely to be benign while the latter is more likely to be malignant.

Recently, Parker showed that the H-scan format for ultrasound can be useful in assessing changes in the concentrations or sizes of small scatterers in liver and placenta tissues.^17^^–^^20^ Briefly, the H-scan analysis uses a matched filter approach to identify Rayleigh (small scatterers) by higher order Hermite polynomials and assigns their echoes to a blue display intensity. Simultaneously, echoes from larger scatterers and interfaces are matched to lower order Hermite polynomial functions and assigned to the red display intensity. In this way, a visual interpretation of the nature of the echoes or backscatter can be viewed and a statistical analysis of the different matched filter channels can also be examined. Specifically, employing a convolution model of pulse–echo imaging of this scatterer,^17^^,^^18^^,^^21^^,^^22^ we assume that a fourth order Gaussian-weighted Hermite (GWH) polynomial designated as a GH4 pulse is transmitted; then this is backscattered, received, and convolved with a GHn matched filter, where, in this discussion for simplicity, n is restricted to even integers, such as GH2, GH4, GH6, and GH8. Thus, the echo e(t) formation model is given as follows:

$$e(t)=TR(\frac{t}{\tau })*bs(t)$$ (1)

and

$$m_{n}(t)=e(t)*GH_{n}(\frac{t}{\tau }),$$ (2)

where the asterisk symbol implies convolution and TR(t/τ) is the round-trip impulse response of the transducer, assumed to be $GH_{4}(t/\tau )$. Furthermore, bs(t) is the impulse response of the scatterer, $GH_{n}(t/\tau )$ is the H-scan channel matched filter assigned to a color, and $m_{n}(t)$ is the output of the matched filter.

For example, the $GH_{2}$ function is defined as follows:

$$GH_{2}(\frac{t}{\tau })=e^{-{\left(\frac{t}{\tau }\right)}^{2}}[4{\left(\frac{t}{\tau }\right)}^{2}-2],$$ (3)

and the Fourier transform of $GH_{2}(t/\tau )$ is

$$I\{GH_{2}(\frac{t}{\tau })\}=\frac{e^{-\frac{1}{4}\tau^{2}\omega^{2}}\tau^{3}\omega^{2}}{\sqrt{2}},$$ (4)

and, in general,

$$I\{GH_{n}(\frac{t}{\tau })\}=\frac{e^{-\frac{1}{4}\tau^{2}\omega^{2}}\tau^{n+1}\omega^{n}}{\sqrt{2}}\;\text{for}\;n\in \text{even integers}.$$ (5)

**Fig. 1:**
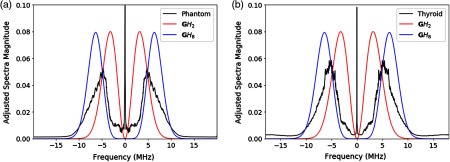
(a) Ensemble averaged spectra of the test phantoms (27 frames) compared with spectra of the GWH functions $GH_{2}$ and $GH_{8}$. (b) Averaged spectra of the thyroid nodules (46 images) compared with spectra of the GWH functions $GH_{2}$ and $GH_{8}$. For each frame, individual A-line spectra were computed and averaged across the frame. The final spectra shown are subsequent averages of all frames.

**Fig. 2:**
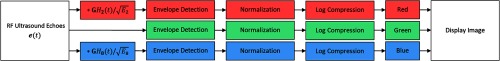
Schematic of the H-scan format. Beamformed A-line echoes collected from a pulse–echo scanner are processed in a parallel fashion. Convolution with $GH_{2}$ is assigned red, convolution with $GH_{8}$ is assigned blue, and the envelope is assigned green. Processing can be done in real time or on stored RF signals. All three channels are typically displayed on a 45-dB dynamic range.

Some examples of spectra are given in Fig. 1. The flow chart of the processing of echoes e(t) is shown in Fig. 2, where the output of the matched filters is assigned to colors in the final display.

We hypothesize that the H-scan analysis will identify different scatterer distributions among benign and malignant thyroid lesions. In this preliminary study, we apply the H-scan format to benign and malignant thyroid nodules to show that differences in scatterers may be helpful in early screening for high-risk groups, in reducing the number of biopsies, in diagnosis, and for potential treatment of thyroid cancers.

## 2 Methods

### 2.1 B-Scan Acquisition

Data acquisition was performed using a SonixTouch ultrasound scanner (Analogic Ultrasound, Peabody, Massachusetts) equipped with an L14-5 linear array transducer (nominal center frequency of 10 MHz) at a sampling rate of 40 MHz. The imaging sequence used focus transmit and dynamic receive over an imaging region of interest (RoI) ∼3.5  cm in depth and 4 cm in width. The corresponding digitized radiofrequency (RF) was comprised of ∼2000 samples axial and 256 lines laterally. Typical transmit beams were produced with a 1-cm aperture focusing at 2-cm depth, and the frame rate was over 10  frames/s. A 45-dB dynamic range on the envelope was used to display the B-scans and the H-scans.

### 2.2 Phantoms

Two test phantoms were constructed to demonstrate the H-scan format’s sensitivity to scatterers of different sizes. Both phantoms consisted of a cylindrical agar background of 9.6 cm diameter, with suspended glass beads (Potter Industries, Valley Forge, Pennsylvania) at a concentration of 4  g/l and a speed of sound of ∼1540  m/s. The first phantom contained scatterers with a narrow diameter distribution ranging from 39 to 43  μm and had an attenuation coefficient slope of 0.1  dB/cm/MHz. The second phantom contained glass scatterers that ranged from 90 to 106  μm in diameter and had an attenuation coefficient slope of 0.5  dB/cm/MHz.

### 2.3 Thyroid

A set of ultrasound images from 46 patients (8 males, 38 females, 55.7±12.5 years old) was acquired in an oncology clinic right before the fine needle aspiration biopsy (FNAB) under the requirements of informed consent and the Pontificia Universidad Católica del Perú Institutional Review Board. All nodules analyzed in this study met the criteria of not exhibiting partially cystic or honeycomb patterns or calcifications and having FNAB results reported as either Bethesda II (benign, $N_{\text{benign}}=30$) or VI (cancer, $N_{\text{maligant}}=16$). The summary of the FNAB results for all patients is reported in Table 1. The data were completely anonymized, with patient labels and identifiers removed. RoIs containing the relevant nodules were identified by clinicians in the corresponding B-mode images and used in the analysis.

**Table 1:** FNAB results for all subjects in this study.

Benign nodules		Malignant nodules	
Adenoma	18	Papillary thyroid carcinoma	13
Hashimoto’s thyroiditis	8	Hurthle cell carcinoma	1
Colloid nodule	3	Follicular variant papillary thyroid carcinoma	2
De Quervain’s thyroiditis	1		

### 2.4 H-Scan Analysis

The received, beamformed raw RF data for both the phantom and thyroid images were used for application of the H-scan format. The ensemble averaged spectra over all A-lines within the RoI of the test phantoms and thyroid nodules compared with the spectra of the GWH functions are shown in Fig. 1. Lower frequencies and larger reflections are captured by the matched filter formed by the GWH function $GH_{2}$. The higher frequency content and Rayleigh scatterers are captured by the matched filter formed by the function $GH_{8}$. The $GH_{2}$ and $GH_{8}$ functions were sampled at 40 MHz, as were the RF echoes, for discrete convolution. The schematic for the H-scan format application is shown in Fig. 2.

The H-scan format was also modified to emphasize echoes from the dominant channel. Each pixel is assigned the greater of either its red or blue value, while the green values are zeroed out, thus producing an image displaying the dominant hue and still within the 45-dB dynamic range.

### 2.5 Statistics

For both the test phantoms and the thyroid nodules, standard parameters, such as the mean pixel values (in magnitude or in dB) of each RGB channel, were measured and compared between appropriate groups (e.g., between test phantoms with different scatterer sizes or between benign and malignant nodules).

However, in an effort to provide more detailed analysis, H-scan outputs from each image RoI within a group were combined to form one group dataset. Thus, metrics in the form of “group statistics” were tested. To acknowledge the fact that pixels are not independent of their nearest neighbors, each image was downsampled according to the autocorrelation widths along both axial (∼0.15  mm) and lateral (∼2.0  mm) directions to be able to apply appropriately the statistical tests on independent samples within the RoIs.

We also examined the pixel-by-pixel difference in the magnitudes of the red and blue channels, corresponding to the difference in matched filter outputs for smaller scatterers (higher frequencies assigned to the blue channels) and larger scatterers (lower frequencies assigned to red). A Welch’s unequal variances t-test was used to test if two groups had different means for this metric. A Kolmogorov–Smirnov test was used to test if the two groups came from different continuous distributions. Differences in the means that were statistically significant would indicate some level of structural differences between the two groups being compared. For the statistics, the following notation is used: ns (no significance); p>0.05; *p<0.05; **p<0.01; ***p<0.001; and ****p<0.0001.

All the analysis for the H-scan format and statistics were performed using MATLAB R2017a (The Mathworks, Inc., Natick, Massachusetts). Figures (except Fig. 2) were generated in Python 3.6.2 (Python Software Foundation, Ref. 23).

## 3 Results

**Fig. 3:**
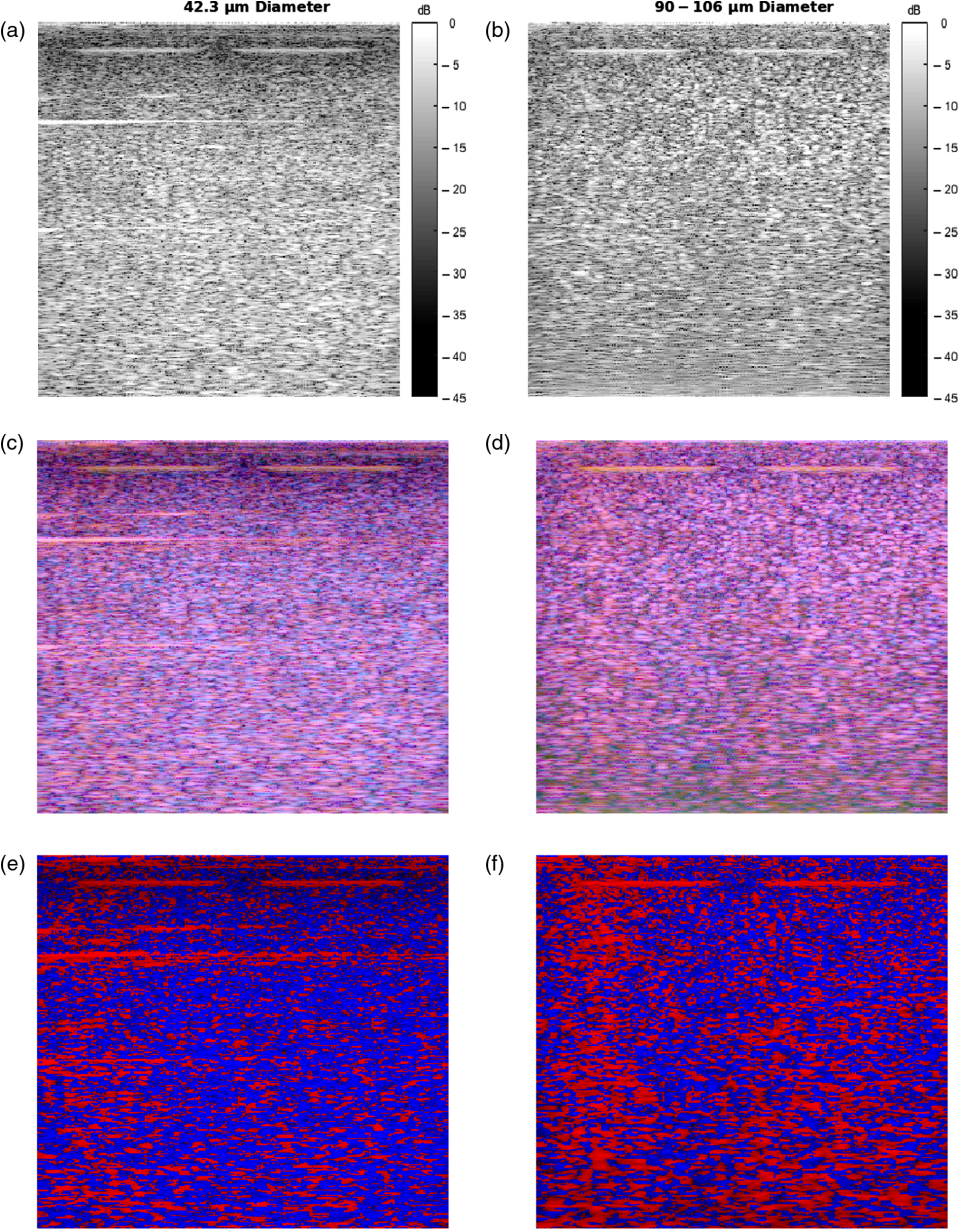
(a and b) B-scans, (c and d) H-scans, and (e and f) modified H-scans of the test phantoms consisting of 42.3-μm diameter scatterers and 90- to 106-μm diameter scatterers. Color bars not shown for H-scans due to triple channels; each RGB channel is displayed according to a 45-dB dynamic range.

Application of the H-scan analysis to the sample test phantom frames is shown in Fig. 3. The H-scans demonstrate obvious visual differences between the phantom frame with 42.3-μm sized scatterers and the phantom frame with 90- to 106-μm sized scatterers. Modified H-scans show the predominant hue, as described in Sec. 2. Qualitatively, the test phantom with larger diameter scatterers exhibits more red intensities in the H-scan, whereas the test phantom with smaller diameter scatterers retains higher blue intensities. These results are consistent with theoretical expectations and serve to verify the methods.

**Fig. 4:**
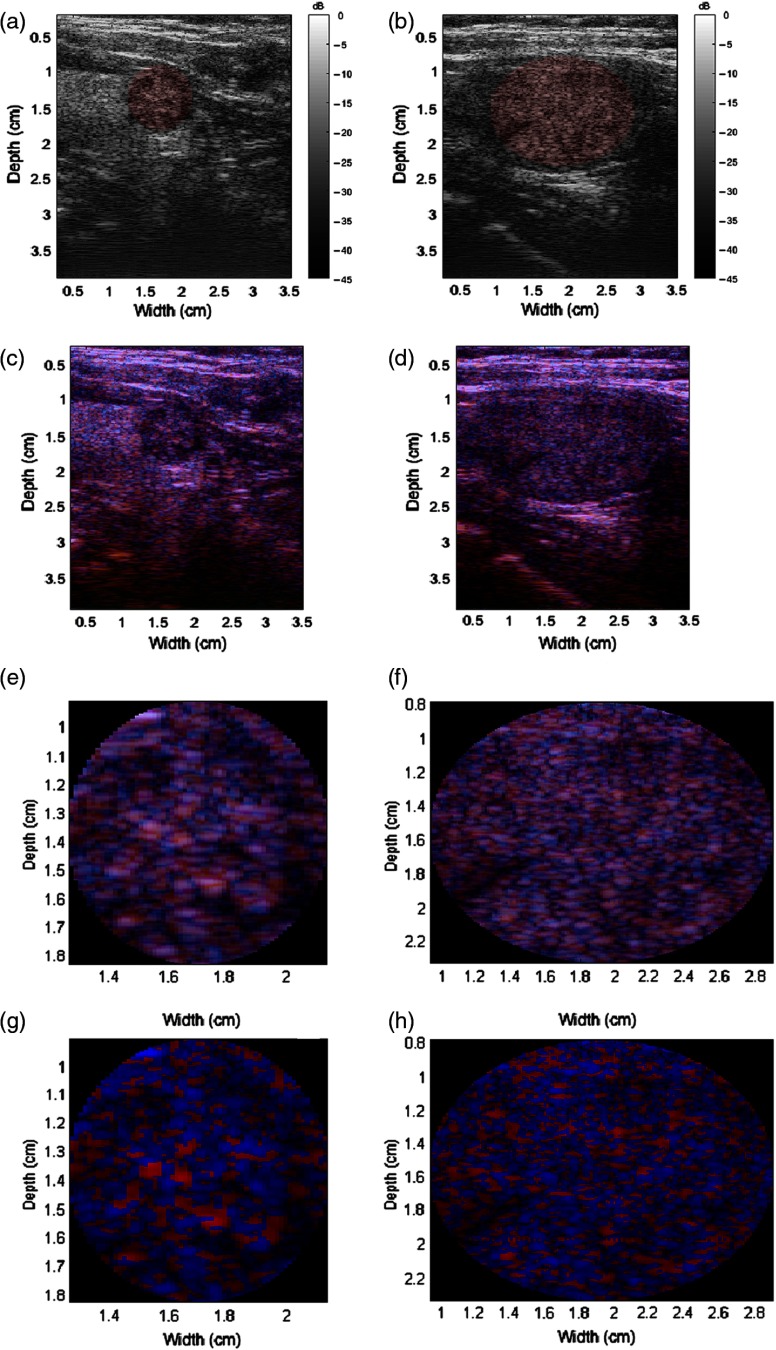
Benign nodule examples. (a and b) B-scans with the RoIs highlighted red, (c and d) H-scans, (e and f) zoomed-in H-scans of the RoIs, and (g and h) modified H-scans of the RoIs. Color bars not shown for H-scans due to triple channels; each RGB channel is displayed according to a 45-dB dynamic range.

**Fig. 5:**
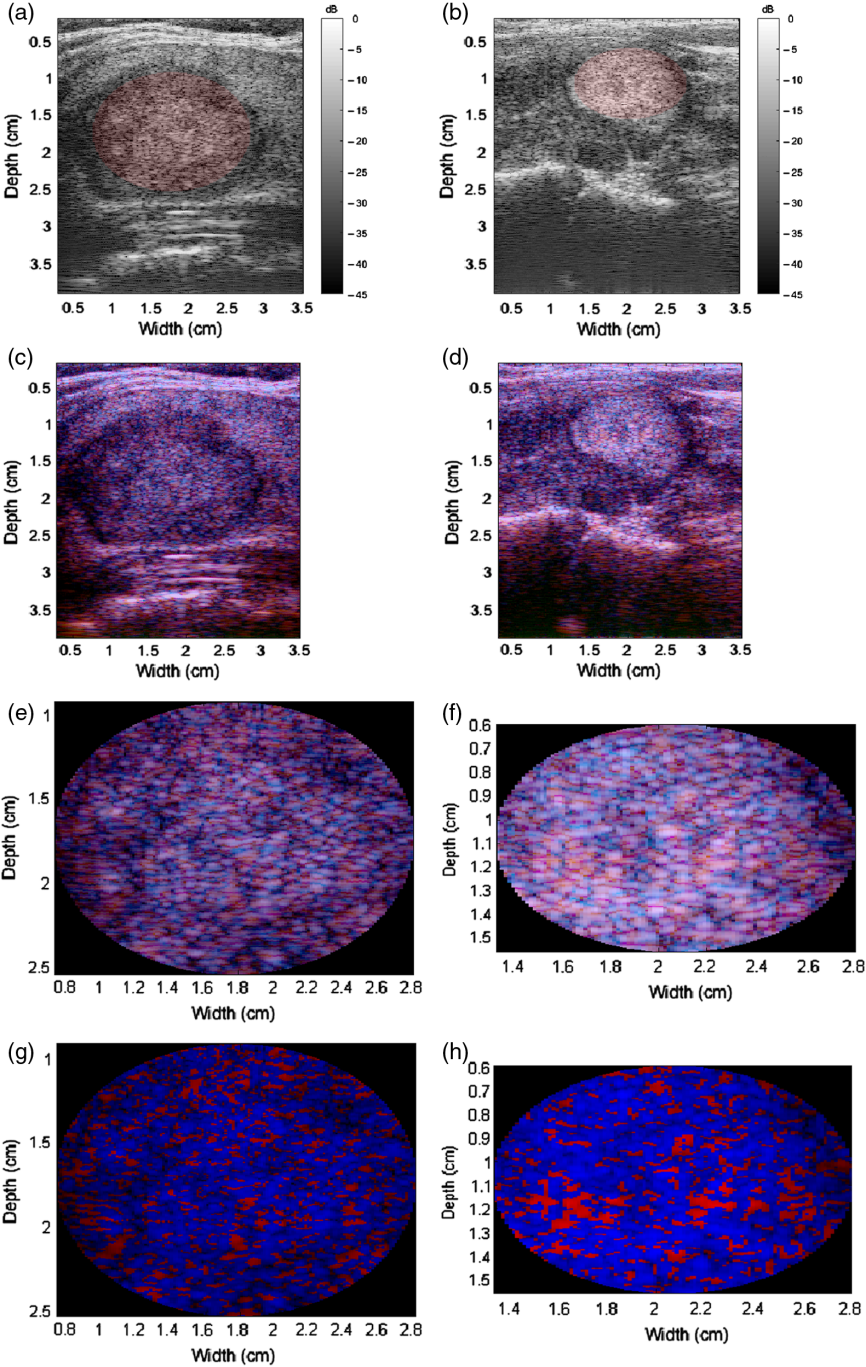
Malignant nodule examples. (a and b) B-scans with the RoIs highlighted red, (c and d) H-scans, (e and f) zoomed-in H-scans of the RoIs, and (g and h) modified H-scans of the RoIs. Color bars not shown for H-scans due to triple channels; each RGB channel is displayed according to a 45-dB dynamic range.

The H-scan format was also applied to all thyroid nodules. Examples of benign nodules are shown in Fig. 4, while examples of malignant nodules are shown in Fig. 5. The malignancy of the nodules was verified with biopsy results. In addition, the RoIs containing the nodules are shown. Qualitatively, the malignant nodules shown demonstrate more blue intensities while the benign nodules contained more red intensities. However, immediate and pronounced color shifts were not readily apparent for all nodules in the dataset.

**Fig. 6:**
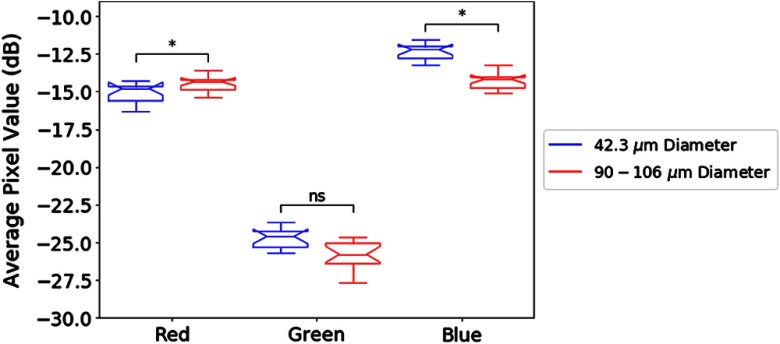
Notched boxplots of the average pixel value (in dB) of each RGB channel between 42.3-μm and 90 to 106-μm scatterer phantoms. Differences in the average pixel values were observed in the red and blue channels between the two test phantoms.

**Fig. 7:**
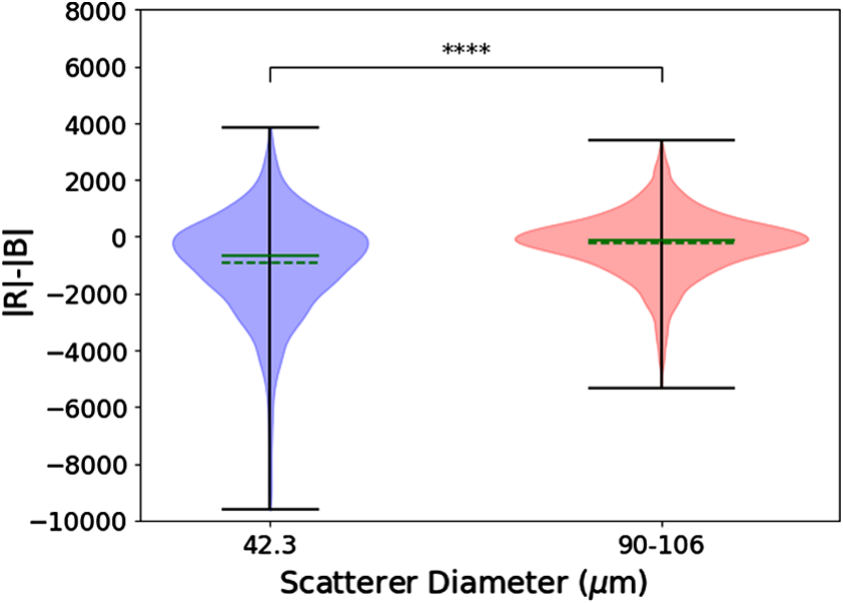
Group statistics of the pixel-by-pixel measure of the magnitude of the red channel minus the magnitude of the blue channel (a test of the relative output of matched filters influenced by scatterer size) for two phantoms, displayed as violin plots. Solid green line = median. Dashed green line = mean. A Welch’s unequal variances t-test indicates that the means are not equal (p<0.001) and a Kolmogorov–Smirnov test indicates that the two groups come from different continuous distributions (p<0.0001).

The statistical methods described in the previous section are applied to the test phantoms and the thyroid nodules. In Fig. 6, a notched boxplot shows statistically significant differences between the mean pixel value among the red and blue channels of the two sets of test phantoms. Figure 7 portrays the distributions of |R|−|B| from each pixel, a measure of the relative strength of the two channels, for the two test phantoms. The distributions have different means (p<0.001) and do not come from the same continuous distributions (p<0.001).

**Fig. 8:**
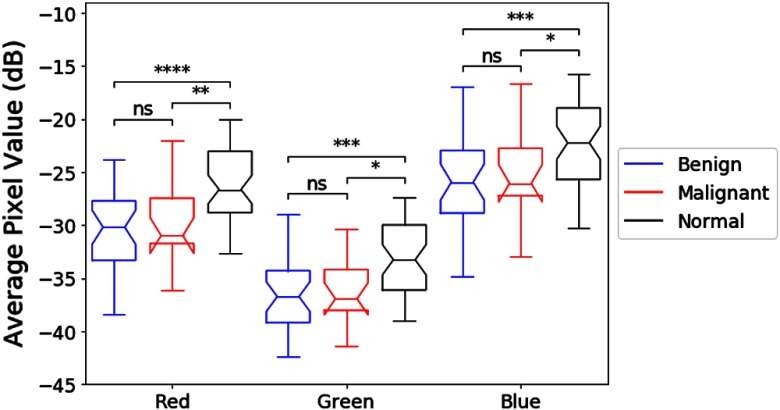
Notched boxplots of the average pixel value (in dB) of each RGB channel of benign RoIs, malignant RoIs, and normal thyroid regions. Differences in the average pixel values were observed in all three channels among the nodules and the normal thyroid regions using analysis of variance (ANOVA) (all channels p<0.001). Bonferroni corrected pairwise t-tests were applied as follow-up multiple comparisons.

**Fig. 9:**
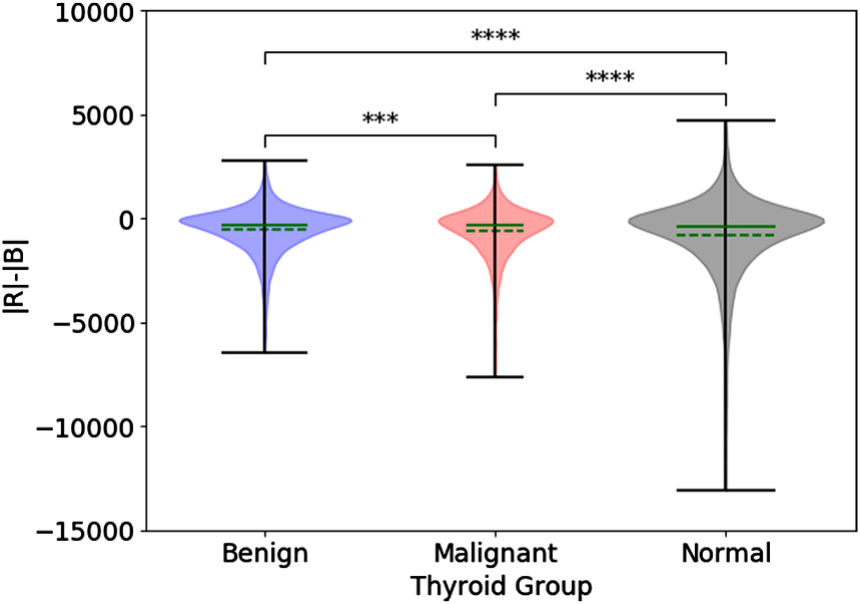
Group statistics of |R|−|B| for all thyroid regions of interest, displayed as violin plots. Solid green line = median. Dashed green line = mean. Significant differences were tested using ANOVA (p<0.0001). Bonferroni corrected pairwise t-tests were applied as follow-up multiple comparisons. Bonferroni corrected pairwise Kolmogorov–Smirnov tests indicate that any combination of two distributions come from different continuous distributions (all p<0.001).

Similarly, for the thyroid nodules, Fig. 8 indicates that there is a statistically significant difference between average pixel values of the red and blue channels among the benign nodules, the malignant nodules, and regions of normal thyroid. However, a posteriori analysis indicates that there is only a weak difference between benign and malignant nodules, not statistically significant. However, differences between the nodules and the regions of normal thyroid exist, indicating that the benign and malignant lesions in this study were, on the average, hypoechoic as compared with normal thyroid backscatter. Figure 9 portrays the distributions of |R|−|B|, as a measure of the relative strength of the two Hermite matched filters, for the benign and malignant thyroid groups. The distributions have different means (p<0.001) and do not come from the same continuous distributions (p<0.001). For Figs. 6 and 8, the statistical differences are preserved with raw pixel magnitude values (without the dB conversion).

## 4 Discussion and Conclusion

The results of the test phantoms verify the utility of the H-scan analysis and demonstrate viability in differentiating different sized scatterers in tissue. The phantom with the smaller scatterers was more blue in hue while the phantom with the larger scatterers was more red in hue, which is in agreement with the theoretical basis of the H-scan analysis.

When the H-scan analysis was applied to the thyroid nodules, a small but statistically significant difference was observed in the |R|−|B| distributions, as shown in Fig. 9. These results suggest a fundamental difference in distributions of the scatterer sizes, albeit one that is more subtle than the case of the two phantoms (Figs. 3, 6, and 7).

A related quantitative measure of the H-scan outputs is obtained by taking the ratio of the average (or median) R output to the average (or median) of the B output over RoIs, and this measure is independent of echogenicity and amplifier gain. In theory, as scatterers or structures become larger in size than the Rayleigh scattering long wavelength limit, then this ratio should increase.^17^^–^^19^ The results for this study are given in Table 2, presented in ascending order. However, these are uncorrected for attenuation. The standard error of the mean for each group is on the order of ±0.06 for RoIs of size ∼1.5  cm×1.5  cm. This progression of R/B ratios suggests that the characteristics of scatterers averaged across the normal thyroid RoIs, and the benign and malignant RoIs, appear as smaller than the 42  μm scatterers using the H-scan analysis, but they require further work to correct for the effects of frequency-dependent attenuation.

**Table 2:** Ratio of uncorrected |R|/|B| in ascending order.

Normal thyroid	0.48
Benign lesions	0.52
Papillary carcinoma	0.54
42-μm spheres	0.61
90 to 102-μm spheres	0.83

An accurate physical model of the thyroid gland and malignancy would provide more useful insights of the results. Quantitative autocorrelation functions derived from pathology slides have been shown to predict scattering functions,^24^^,^^25^ and this approach could be helpful for modeling the scattering from thyroid pathologies. Despite some ambiguity, the H-scan format may still prove to be useful clinically for its qualitative characteristics. As shown with both the test phantoms and the thyroid nodules, rendering of colored images can provide clinicians additional information about scatterer classes. The clinical utility would have to be demonstrated by improvement in measurable patient outcomes.

There are a number of limitations of this study that circumscribe the results. An important set of these pertain to the limitations of the scanning system and bandwidth, which restrict the separation of the GWH matched filters and our ability to discriminate between shifts in scattering types. The system produces a nominal peak near 5 MHz, but the antialiasing filter and the effects of frequency-dependent attenuation create a dramatic reduction of signal strength at higher frequencies. Thus, our $GH_{8}$ channel matched filter, assigned to blue, is more highly diminished by attenuation as compared with the effects on $GH_{2}$. As attenuation accumulates with depth, these ratios are depth-dependent. These two effects have not been compensated in this study, and future work is needed to address this and improve the full bandwidth. In addition, no special modifications were made to the transmit pulse excitation to improve its conformity to a GWH polynomial. Future work on this and on the compensation for frequency-dependent attenuation, along with other system improvements for improved bandwidth, will better optimize the H-scan analysis for subtle shifts in scattering within lesions.

Within the limits of the current system, the study suggests that analysis of different sized scatterers can be helpful in predicting malignancy of thyroid lesions. The results indicated that there are subtle but statistically significant differences between benign and malignant nodules, and more robust methods should be developed to emphasize the differences found. Furthermore, the H-scan analysis may also prove useful in studying the structural properties of the thyroid gland, particularly when malignancy is involved to provide further insight on the disease process.
